# Haptic Categorical Perception of Shape

**DOI:** 10.1371/journal.pone.0043062

**Published:** 2012-08-10

**Authors:** Nina Gaißert, Steffen Waterkamp, Roland W. Fleming, Isabelle Bülthoff

**Affiliations:** 1 Max Planck Institute for Biological Cybernetics, Tübingen, Germany; 2 University of Giessen, Giessen, Germany; 3 Department of Brain and Cognitive Engineering, Korea University, Seoul, Korea; Queen Mary University of London, United Kingdom

## Abstract

Categorization and categorical perception have been extensively studied, mainly in vision and audition. In the haptic domain, our ability to categorize objects has also been demonstrated in earlier studies. Here we show for the first time that categorical perception also occurs in haptic shape perception. We generated a continuum of complex shapes by morphing between two volumetric objects. Using similarity ratings and multidimensional scaling we ensured that participants could haptically discriminate all objects equally. Next, we performed classification and discrimination tasks. After a short training with the two shape categories, both tasks revealed categorical perception effects. Training leads to between-category expansion resulting in higher discriminability of physical differences between pairs of stimuli straddling the category boundary. Thus, even brief training can alter haptic representations of shape. This suggests that the weights attached to various haptic shape features can be changed dynamically in response to top-down information about class membership.

## Introduction

Categorical perception (CP) was first observed for color [1] and speech perception [2] and can be described easily using the example of a rainbow. Although the rainbow consists of a continuous spectrum of different wavelengths, human perception parses this continuum into a limited number of distinct color bands (e.g. blue or green) [1]. Thus, CP describes a phenomenon where stimuli varying gradually along a physical continuum lead to a small number of discrete perceptual categories [3]. This phenomenon is further characterized by the fact that equal-sized physical differences between stimuli are perceived as larger or smaller depending on whether the stimuli belong to the same or to different categories [4]. Thus, two color shades are perceived as being more similar if both are labeled as yellow than if they are labeled as yellow and green even though both pairs of stimuli are equally spaced in terms of wavelength. In other words: CP is characterized by between-category expansion and/or within-category compression in terms of perceived similarity. Further it gives rise to a boundary effect that exaggerates transitions between different categories. The boundary effect itself is characterized by a heightened discriminability for a stimulus pair when the stimuli belong to two different categories and a reduced discriminability for a stimulus pair when the stimuli belong to the same category [3], [5]. Returning to the example of the rainbow, although the wavelengths vary continuously, we perceive a series of distinct transitions or ‘steps’ between the different color bands, which are perceived as steeper than the changes within each band. These key effects (between-category expansion, within-category compression and increased discriminability at category boundaries) are the hallmarks of CP.

Categorical perception is considered an important phenomenon because it demonstrates a powerful top-down effect in perception. When CP occurs, stimuli are not only assigned to categories, but the mental category structure actually alters the perceptual representation of the underlying physical dimensions. For example, color categories distort the perceptual relationship between different wavelengths in color perception [1], and phoneme categories distort the perceptual representations of voice onset times in speech [2]. Thus, importantly, CP is not simply the process of learning categories or concepts. In order for CP to be said to occur, the underlying perceptual dimensions must be systematically altered, increasing the discriminability of stimuli that cross category boundaries.

A number of studies have revealed CP in more complex stimuli, e.g. music [6], rhythm [7], material [8], shape [9] and even faces (e.g., [10]–[12] among many others). Thus, CP seems to be a general principle governing perception, which may be related to how neural networks represent categories in the human brain [3]. Nevertheless, all these studies so far have been restricted to the visual and auditory systems. Here, we show that CP effects can also occur in the haptic modality. We generated a set of tangible objects spanning a physical shape continuum and performed classical classification and discrimination tasks to look for a quantitative discontinuity in discriminability at the category boundary.

Haptic experiments with complex shapes are technically challenging and are also time consuming for participants to perform. For this reason relatively few studies have analyzed how humans categorize objects haptically (compared to equivalent experiments in the visual modality). Early work on haptic object recognition [13] demonstrated that haptic exploration can support rapid and accurate recognition of everyday objects. More recently, many studies have compared shape perception and categorization performance across visual and haptic modalities (e.g., [14]–[17]), and studied visual-haptic integration (e.g., [18]–[20]). Other experiments have shown that haptic recognition and classification performance is sensitive to the orientation of the object relative to the training orientation, similar to the viewpoint sensitivity found in visual object recognition [21]–[23]. A number of other groups have studied neural representations of haptic shape encoding [24], [25].

Lederman & Klatzky [26] analyzed how humans categorize common, everyday objects, while Haag [27] compared visual and haptic categorization of toy objects, which resembled miniaturized animals. While these studies have shown that humans are impressively good at haptic recognition and classification of familiar objects, such stimulus sets have a number of limitations. First, they are hard to characterize in terms of important object parameters such as shape or texture. Second, using familiar objects means that subjects enter the experiment with the categories already in place. This means it is not possible to test performance before and after training to measure the effects of the learning process. Third, having recognized a given object, subjects can use semantic knowledge, perhaps acquired via other senses, to influence their judgments (for a recent review about that subject for color perception, see: [28]).

In contrast, Schwarzer [29], Cooke [15] and Homa [30] and their colleagues used fully controlled, novel stimuli and thus participants had to base their categorization behavior solely on object-intrinsic properties, rather than pre-established semantics. As these studies show, the haptic sense is not only suitable for correctly categorizing familiar every-day objects, but can also categorize novel objects. This categorization behavior was shown to be influenced by task [31] and training [32]. These studies have shown that objects within a category are perceived to be more similar than objects from different categories. However, this raises the question: what happens at category boundaries? Despite substantial progress in our understanding of haptic category formation, previous studies have not revealed a CP effect, because they did not test for an increase in discriminability at the category boundary.

By contrast, here we explicitly tested for changes in the discriminability of different shapes brought about by category learning. It is important to note that there are good reasons for expecting discrimination performance *not* to be affected by class membership in any sensory modality. In the case of haptic shape perception, it could be that discrimination performance is driven by low-level feature differences between the objects, irrespective of the class to which the object is assigned. For example, labeling an object as belonging to class A does not affect the physical curvatures, texture or size of the object, which could serve as the basis for discrimination. Despite this, we find that a surprisingly small amount of training can lead to clear changes in haptic shape representations, consistent with the criteria for CP. We argue that shape is represented as a weighted combination of many mid- and high-level features, and that the weights attributed to different features can vary dynamically in response to top-down information about class membership.

**Figure 1:**
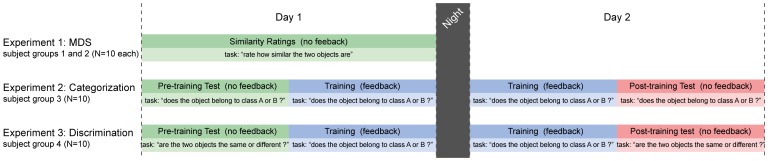
Overview of experiments. Experiment 1 (MDS) was designed to measure the perceptual uniformity of the space of stimuli used in the other experiments in one session. Experiments 2 (Categorization) and 3 (Discrimination) were conducted in two sessions on consecutive days. The first session consisted of pre-training testing, followed by training with feedback. The second session consisted of top-up training followed by a post-training test. We compare performance before and after training to measure the effects of category learning on the ability to categorize untrained stimuli (Experiment 2) and discriminate between stimuli in a same-different task (Experiment 3).

In order to test for CP, we manufactured a set of objects that vary continuously in shape between two prototype objects (A and B). We then ran three experiments with different groups of participants to measure category learning and its effects on discrimination performance (summarized in Figure 1). First, in Experiment 1, we used multidimensional scaling (MDS) to confirm that the stimuli were roughly evenly spaced in terms of their *a priori* perceptual similarity. This allows us to ensure the stimuli are suitable for measuring CP effects in the other two experiments.

Then, in Experiment 2, we used a test–training–re-test design to measure category learning. The purpose of this experiment was to demonstrate that the training is effective at teaching participants categories A and B. Specifically, we first presented participants with objects A and B and then tested their initial ability to categorize the intervening objects as belonging to class A or B. This establishes a baseline ability to classify intervening objects. Then we trained the participants with explicit feedback on a *different* set of intervening objects until they could consistently categorize them as belonging to either class A or B. Finally, we re-tested their ability to categorize the original set of intervening stimuli after training, to confirm that the training teaches participants the categories. Using a different subset of the intervening stimuli in the test and training sessions ensures participants learn the concepts A and B, rather than just learning to recognize the specific exemplars. If participants learn the classes A and B, we expect an improvement in categorization performance after training. Note that this category learning process is a pre-requisite for CP, but does not, on its own, demonstrate CP.

Finally, in Experiment 3 we used a discrimination task to measure the effects of category learning on the participants’ ability to discriminate the different stimuli. A new set of participants again performed a test–training–re-test regime, except that instead of reporting which class each stimulus belonged to, they had to discriminate between objects in a same-different task. If CP does not occur, we would expect the training not to significantly impact their ability to discriminate between similar shapes in the re-test phase compared to the initial (pre-training) test phase. By contrast, if CP does occur, we would expect to see an increase in the ability to discriminate stimuli that flank the category boundary following training, demonstrating a top-down effect of category assignment on the perceptual representation of haptic shape features.

## Methods

### Ethics Statements

The research presented here consists of standard haptic tasks with seated participants palpating plastic objects. This falls under standard testing procedures for research in non-public institutions that do not involve drugs, and therefore did not require any specific ethics approval from the ethics review board. All experiments were conducted in accordance with the 1964 declaration of Helsinki. Participants signed a general consent form stipulating that they agree to have their data used anonymously and published and they were informed of their right to remove their data at any time. Before the start of each experiment session, informed, oral consent was obtained from all participants about the specific experiment. Participants were informed that they could stop at any time. All data was kept and analyzed anonymously. An experimenter was present at all time during the experiments.

### Participants

Forty right-handed participants (age: 21 to 61 years) completed the experiments, in four distinct groups of 10 participants each. Group 1 (Experiment 1, front ‘view’): 4 female. Group 2 (Experiment 1, rear ‘view’): 4 female. Group 3 (Experiment 2): 5 female. Group 4 (Experiment 3): 5 female. No significant gender differences were found, so none are reported in the results.

### Stimuli

**Figure 2:**
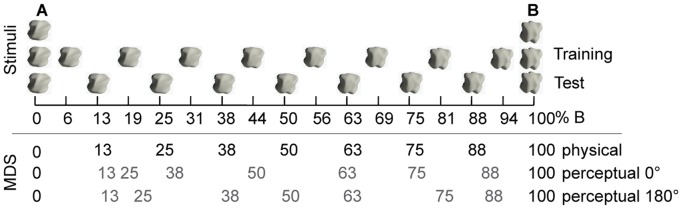
Stimuli. The top row shows the two prototypes A and B used to create all intervening objects. The second and third rows represent the 15 training and test objects, respectively. Note that training and test sets differ, except for the prototypes, which were used in all sessions. The x-axis displays the proportion of B-features in each intermediate (morph) object, as a percentage. The bottom three rows show the MDS results for two object orientations (0° and 180°, see text for more details). The numbers in black (top row) indicate the relative positions of the physical stimuli in terms of the percentage of B features. The numbers in grey (bottom two rows) indicate the perceived differences between the test stimuli. Specifically, the horizontal position of each grey number along the morph line indicates the perceived position of the corresponding stimulus, as calculated by MDS. The perceptual MDS maps were linearly scaled to the same range as the physical stimuli, for easy comparison. It is clearly visible that participants perceive the morphed objects to span a roughly equally-spaced morph line independent of object orientation (10 participants were tested for each orientation). Note that the perceived ordering of the stimuli is preserved.

To generate tangible objects, we combined computer graphics modeling with rapid 3D prototyping. Two objects, A and B, were generated using the 3D modeling software 3D Studio Max by taking a sphere of 7 cm diameter and overlaying it with two wave modifiers each, resulting in roundish objects with small hills and valleys. These two objects were then morphed into each other and 15 intermediate morph objects were generated at equally-spaced morph steps. The objects were then printed using a 3D printer (ZPrinter 650, ZCorporation, Germany). All objects were equal in weight and volume and were mounted on small stands for easier haptic exploration. The final stimulus set consisted of 17 different shapes (see Figure 2). Since the experiments consisted of training and testing conditions, we split the stimuli into a training set and a test set to ensure that participants learnt category features rather than individual objects. The training and test sets were interdigitated equally along the morph continuum (see Figure 2). As all objects are related to each other, a common arbitrary base viewing point (0 degree) could be defined for all of them and was used for orientating the objects relative to the observers in the experiments. Throughout all experiments, the stimuli were freely explored by blindfolded, right-handed participants.

### Experiment 1: MDS

To ensure that the stimulus set was suitable for identifying categorical perception, we performed similarity ratings and multidimensional scaling (MDS) analyses to verify that participants were able to discriminate objects A, B and the intervening morphed objects, and that participants perceived the objects to span a roughly equally-spaced sequence.

Since haptic experiments are very time consuming we only used the nine stimuli of the *test set* for the similarity ratings. The task was to rate the similarity between pairs of objects on a scale from low similarity (1) to high similarity (7). Blindfolded participants freely explored the nine objects with their right hand. The exploration time was restricted to six seconds. Every object was compared once to itself and once to every other object resulting in 45 object pairs. All object pairs were shown in randomized order in one block. Participants had to perform three blocks in total and were allowed to take a break between blocks. Ten participants palpated the objects in 0° orientation, ten others in 180° orientation (groups 1 and 2 in Figure 1), to ensure that there were no orientation-specific artifacts in the objects (The 0 deg orientation was chosen arbitrarily for object A and that orientation and by extension the 180 deg orientation and the orientations in-between were defined thereby for all other objects). By using these two orientations and free object exploration, we covered the whole 3D shape of the objects [21]. The similarity ratings were averaged across participants and analyzed using non-metric MDS (MDSCALE in Matlab see [17] for a detailed description on how to perform and analyze MDS of complex parametrically-defined objects in combination with haptic object exploration). The MDS output map is visualized for a one-dimensional solution in Figure 2. The physical spacing between the different numbers indicates the distances returned by MDS between the corresponding stimuli. The figure shows that objects A and B are clearly distinguishable, that the morphs in between objects A and B are also distinguishable and perceived in correct order, and furthermore that they are almost equally spaced perceptually (we tested this also for two- and three-dimensional MDS output maps and found the same results). Thus the stimulus set is well suited for identifying CP effects.

### Experiment 2: Classification

The experiment consisted of (1) a pre-training test, (2) a training phase and (3) a post-training test. Because of time constraints the experiment was divided into two sessions, which took place on two consecutive days. The first session started with a test phase, followed by a training phase. The second session started with a training phase to verify that the training consolidated during the night, and was followed by the post-training test. Ten new participants (group 3) conducted both sessions. They explored one object at a time using their right hand and had 4 seconds to explore the object freely.

The pre-training test started by introducing the participants to objects A and B. Object A was presented in orientations 0°, 60°, 120°, 180°, 240° and 300°. Then, B was presented in the same orientations. Next, the seven morph-objects of the *test set* were presented in random order in one of the six orientations selected arbitrarily (we ensured that every orientation of every object occurred at least once). Participants had to say whether the object belonged to category A or B. No feedback was provided. The testing was repeated ten times for each object. After half of the test trials, A and B were presented again from all six orientations as a reminder.

Next, participants had to pass the training. The training was similar to the pre-training test, except that feedback was provided, and we used the *training set* to ensure that participants learned the category-related features rather than the objects themselves. Participants again indicated whether the presented object belonged to category A or B. Training ended when participants correctly classified at least 7 out of 8 objects, three times in a row.

On the following day, participants had to perform the training and reach the learning criterion again before conducting the post-training test. The post-training test exactly was identical to the pre-training test.

### Experiment 3: Discrimination

**Figure 3:**
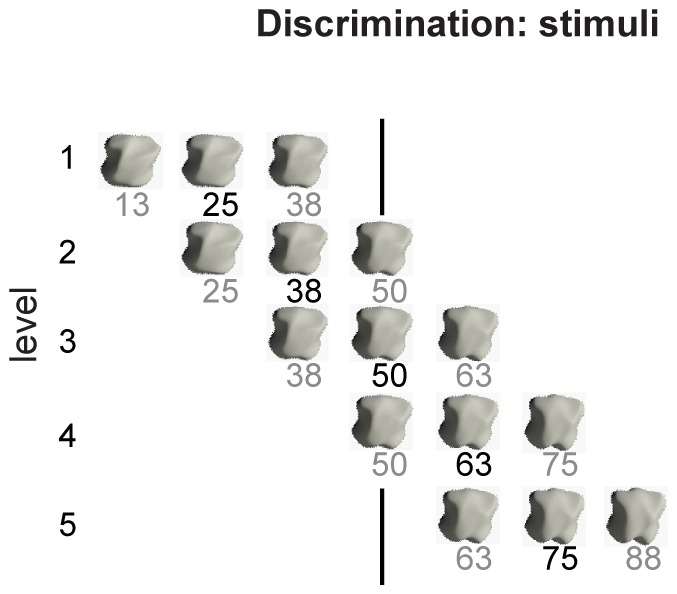
Discrimination experiment: stimuli and trials. Representation of the five *object levels* used in the discrimination experiment. Each level consisted of two trials (one “same” and one “different” trial). The first row shows the objects used for the trials of *object level 1*. Stimulus 25 was shown twice in the “same” trial while the adjacent objects (13 and 38) were shown in the “different” trial. Rows 2 to 5 show the objects used for in the object levels 2 to 5 respectively. In all levels, the object with the number in black was used twice in the “same” trials, while the objects labeled in grey were used in the “different” trials. Note that for *object level 3*, the objects of the “different” trial straddle the physical category boundary.

CP effects are said to occur when object pairs that straddle the category boundary are easier to discriminate than object pairs lying within one category. Standard procedure for testing this effect is to calculate d’. Therefore “same” and “different” object pairs had to be selected. Again, since haptic experiments are very time consuming, only comparisons around 5 morph levels were made. For each level, the “same” pair consisted of one object used twice, while the “different” pair consisted of its neighboring objects. For example, for *level 1*, a “same” trial consisted of object 25 versus object 25, while a “different” trial, consisted of object 13 versus object 38, which are adjacent to object 25 in the test set. Same-different judgments were similarly made for objects having morph values 38, 50, 63, 75, and 88 (see Figure 3).

The experiment consisted of two sessions, with a pre-training test and a learning phase on the first day, and a learning phase and post-training test on the second day. Ten new participants performed this experiment (group 4 in Figure 1). Within the pre-training phase, each object pair was presented four times. Since we had five “same” pairs and five “different” pairs this resulted in (5×5)×4 = 40 pairs. These 40 object pairs were presented in random order and random orientation. In each trial, the participant could freely explore one object for 4 seconds with the right hand, then the object was replaced by the next one which was either the same or a different object and after palpating the second object, participants had to respond “same” or “different”. For the first object, one of the six possible orientations was pseudo-randomly selected. The second object was then presented in the same orientation. Thus, there was no orientation difference between the two objects; the differences that participants perceive were only a result of the morphing process. No feedback was provided.

After this pre-training test, participants went through a training phase. The training was performed in exactly the same manner as in the classification task (see above).

On the following day, participants again completed the same training, followed by the post-training test, which was conducted in exactly the same manner as the pre-training test described in this section.

## Results

### Classification

The experiment consisted of a pre-training test and a training phase on day one and a training phase and the post-training test on day two. To reach the learning criterion participants had to classify at least 7 out of 8 objects correctly in three consecutive blocks. On day one, participants needed on average 8 blocks to reach criterion while they only needed 5.3 blocks on the second day. Thus participants were significantly better on the second day (Wilcoxon signed rank test, *p* = .047, *T* = 1.5, *r* = −.296, effect size *W* = .67) showing that they formed a category representation that was consolidated during the night.

**Figure 4:**
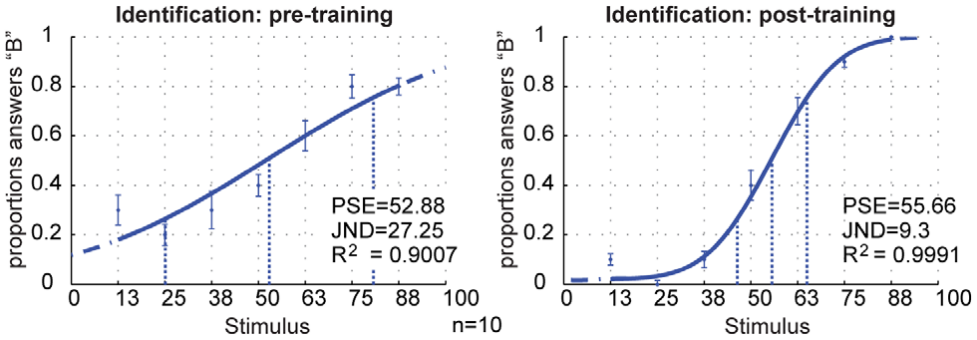
Results of the classification experiment. Left: pre-training; right: post-training. We counted how often every stimulus was identified as object B. These proportions were averaged across participants. Then a cumulative Gaussian was fit to the data. From this sigmoidal function the PSE and JND can be retrieved. Participants categorize A and B correctly before training, but the morphed objects are perceived as a smooth transition. After training the curve is significantly steeper, indicating that training lead to a sharpened categorical boundary. The blue dotted lines indicate the locations on the shape continuum of the 25%, 50% and 75% categorization values. Error bars  =  SEM.

Since ten participants identified every object ten times, once in the pre-training test and once in the post-training test, every object was identified 100 times in each test. We calculated how often every object was identified as object B and plotted the proportion of answers “B” (see Figure 4). A cumulative Gaussian was fitted through these data points using the psignifit toolbox version 2.5.6 for Matlab, which implements the maximum-likelihood method described by [17]. From this sigmoidal function the point of subjective equivalence (PSE) and just noticeable difference (JND, calculated as the difference between the upper threshold which was set to 75% and the PSE) can be retrieved. The steepness of the curve is described by the JND, the lower the JND, the steeper the curve. Perfect classification would yield a step-like function in which all objects with less than 50% B-features would be identified as A and all objects with more than 50% B-features would always be identified as B while object 50 would be arbitrarily assigned to either A or B. Thus, the PSE would be marked by object 50.

Figure 4 shows that the PSE is actually located near object 50 before and after training. Furthermore, participants could already discriminate between objects A and B and also between the morphs, before the training. However, cursory visual inspection of the classification curve does not reveal a sharp step at the category boundary. After training, the curve is much steeper and the JND greatly decreases (from JNDpre = 27.25 to JNDpost = 9.3). The steep, step-like function after training shows that training lead to the formation of category representations. Comparing JNDs of single subject data before and after training shows that this categorical effect is highly significant (Wilcoxon signed rank *p* = .002, *T* = 0, *r* = −.845, effect size *W* = .89). The JND decreased significantly for all participants. Thus, every single subject was able to form a category representation.

### Discrimination

CP effects are characterized by a higher discriminability of inter-stimulus differences for stimuli straddling the perceived categorical boundary as defined in the classification task. Standard procedure to test for this effect is calculating d’, which is the ratio between hit rate (correctly identified “same” or “different” pairs) and the false-alarm rate. Thus, CP should lead to a peak in the d’ value for the object pair straddling the categorical boundary (between-category expansion).

**Figure 5:**
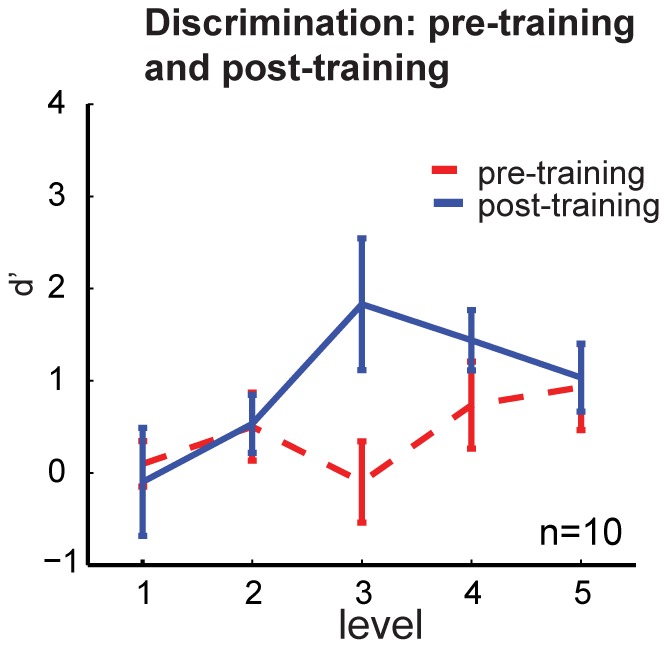
Results of the discrimination experiment. d’ before and after training at every object level (described in Figure 3). Learning increases the discriminability, for *object level 3* and *object level 4* that straddle the perceived category boundary. Error bars  =  SEM.

We calculated d’ for five different equidistant *levels* (see Figure 3) and plotted the values for the pre-training test and the post-training test (Figure 5). Before training, all object pairs are roughly equally discriminable (Friedman test: **χ** ^2^(4) = 2.71, *p*>.05). However, training on shape categories changes the performance. *Level* 3 has the highest d’ value and thus is discriminated most easily after training, followed by *level* 4. As Figure 3 highlights, *level* 3 straddles the physical boundary of the morph-line between object A and B. Performing a Friedman test reveals that the levels are significantly different in discriminability after training (**χ** ^2^(4) = 10.08, *p* = .03. However, post-hoc Wilcoxon tests show that only *level* 4 in comparison to *level* 1 and 2 (*p* = .02, *T* = 0, *r* = −.49, effect size *W* = .75, *p* = .04, *T* = 7, *r* = −.46, effect size *W* = .67 respectively) reveal significant differences. All other *levels* fail to reach the significance level of *p* = .05. This means that the perceived categorical boundary is not located exactly at object 50 but shifted somewhat toward object B.

This finding is consistent with the results of the classification experiment. Figure 4 shows that both before and after training, participants perceive the categorical boundary to be located right of object 50 (*PSEpre*  = 52.88, *PSEpost*  = 55.66). In sum, the results of the classification task indicate that the categorical boundary between object A and B is located slightly to the right of object 50 and that we find between-category expansion after training, i.e. an increase in discriminability for this region.

## Discussion

The ability to learn and generalize perceptual categories from a finite number of exemplars is a crucial faculty of human cognition. In some cases, such as with phoneme or color classes, the mental category structure not only allows us to identify which class a given stimulus belongs to (categorization), but actually alters the underlying perceptual representation of the relevant physical dimensions, exaggerating perceived differences between stimuli that span category boundaries (categorical perception effects), leading to an increase in the ability to discriminate these stimuli. Importantly, learning to label stimuli as belonging to a given class does not necessarily distort the underlying perceptual features in this way, but when it does, this is known as categorical perception.

In the series of studies presented here, we found such CP effects in haptic shape perception. Combining computer graphics modeling with 3D printing, we generated a set of complex objects whose shape features varied continuously in a controlled way. These objects were palpated freely by blindfolded participants in a classification and in a discrimination task. The classification results showed that participants were able to discriminate object A and B prior to learning, however they perceived the intermediate shapes as a smooth transition between the two categories. After learning, the sigmoidal function became significantly steeper, indicating that training was effective as participants became more attuned to category-relevant features of the objects. Furthermore, the JND decreased significantly, indicating that participants were able to perceive smaller differences between objects after training. Finally, the discrimination experiment shows that after learning, participants demonstrated an increased ability to discriminate shape differences around the categorical boundary, a hallmark of CP [3].

In the classification task the perceived category boundary was shifted slightly away from the physical boundary. The large variance partially obscures the location of the performance peak in the discrimination task. In visual or auditory experiments it would be possible to locate the categorical boundary and define the peak more precisely by generating new stimuli and having more trials. However, this was not possible here because of the nature of haptic stimuli and because of the difficulty and length of the haptic experiments (3 hours per participant were needed for Experiment 1, and 4 hours total for Experiment 2 and Experiment 3 each). In our opinion, in the experiments presented here we found a good compromise between accuracy demands and attentional and perceptual limitations of human participants as, despite the limited number of stimuli and trials, there are enough sample points to uncover CP effects reliably after training.

CP effects were first discovered in color and speech perception. In the meanwhile it was shown that CP effects can be induced by learning [4], [9] and were observed for more complex stimuli e.g. material [8], shape [9] and faces [10]–[12]. Although concept learning has been studied in the haptic modality (as reviewed in the Introduction), to our knowledge, this is the first time that CP (*i.e.*, a top-down remapping of perceptual representations following category learning, leading to increased discriminability at category boundaries) has been reported for the haptic modality. Recent advances in computer graphics modeling and rapid 3D prototyping finally allow us to generate adequate stimuli to successfully test for CP in this field.

Together our findings demonstrate that the brain can rapidly alter the way it represents haptic shape features in response to top-down information about category membership. We suggest that the changes do not occur at the level of somatosensory receptors, as these do not measure shape properties directly. During palpation, 3D shape must be inferred from patterns of activity across receptor [33], which must be integrated over space and time, taking into account the relative positions of the digits. Little is known about how this occurs. However, the speed with which category learning leads to CP effects suggests that shape is represented as a weighted combination of a large number of mid- and high-level features extracted from constellations of lower level activity. This is analogous to the idea that visual object recognition relies on mid- and high-level features learned from statistical relations between lower-order image measurements [34], [35]. In order to learn categories of objects haptically, the brain could dynamically re-weight the importance of the different features, leading to the changes in similarity and discriminability we observe in our results (see computational modeling of these processes in [36]).

Relatively little is known about the precise brain mechanisms underlying category formation. Event-related potentials and fMRI data suggest that visual and auditory CP effects evoke activity within the prefrontal cortex (e.g. [37], [38], respectively). Whether there are many modality specific networks or whether one multimodal neural network processes CP effects from all different sensory inputs needs to be determined in future research.

To sum up, in this paper we showed in a classification and a discrimination experiment that category learning of complex shapes can induce CP effects in the haptic modality, and thus that CP is a general principle of perception that occurs across modalities.

## References

[1] Bornstein MH (1987) Perceptual categories in vision and audition. In: Harnad S, editor. Categorical Perception: The Groundwork of Cognition. New York, US: Cambridge University Press. 535–565.

[2] Liberman AM, Harris KS, Howard S, Griffith BC, Hill M (1957) The discrimination of speech sounds within and across phoneme boundaries. Journal of Experimental Psychology 54: 358–368. 13481283 10.1037/h0044417

[3] Harnad S (1987) Psychophysical and cognitive aspects of categorical perception: a critical overview. In: Harnad S, editor. Categorical Perception: The Groundwork of Cognition. New York, US: Cambridge University Press. 1–28.

[4] Goldstone RL (1994) The role of similarity in categorization: providing a groundwork. Cognition 52: 125–157. 7924201 10.1016/0010-0277(94)90065-5

[5] Pastore RE (1987) Categorical perception: Some psychophysical models. In: Harnad S, editor. Categorical Perception: The Groundwork of Cognition. New York, US: Cambridge University Press. 29–52.

[6] Howard D, Rosen S, Broad V (1992) Major/Minor Triad Identification and Discrimination by Musically Trained and Untrained Listeners University of York. Music Perception 10: 205–220.

[7] Desain P, Honing H (2003) The formation of rhythmic categories and metric priming. Perception 32: 341–365 doi:10.1068/p3370. 12729384 10.1068/p3370

[8] Pepperberg IM (1987) Acquisition of the same/different concept by an African Grey parrot (Psittacus erithacus): Learning with respect to categories of color, shape, and material. Animal Learning & Behavior 15: 423–432.

[9] Livingston KR, Andrews JK, Harnad S (1998) Categorical perception effects induced by category learning. Journal of Experimental Psychology-Learning Memory and Cognition 24: 732–753. 10.1037//0278-7393.24.3.732 9606933

[10] Beale JM, Keil FC (1995) Categorical effects in the perception of faces. Cognition 57: 217–239. 8556842 10.1016/0010-0277(95)00669-x

[11] Calder AJ, Young AW, Perrett DI, Etcoff NL, Rowland D (1996) Categorical Perception of Morphed Facial Expressions. Visual Cognition 3: 81–117 doi:10.1111/j.1467–9450.2005.00478.x.

[12] Levin DT, Beale JM (2000) Categorical perception occurs in newly learned faces, other-race faces, and inverted faces. Perception & Psychophysics 62: 386–401 doi:10.3758/BF03205558. 10723217 10.3758/bf03205558

[13] Klatzky RL, Lederman SJ, Metzger VA (1985) Identifying objects by touch: an “expert system”. Perception & Psychophysics 37: 299–302. 4034346 10.3758/bf03211351

[14] Norman JF, Norman HF, Clayton AM, Lianekhammy J, Zielke G (2004) The visual and haptic perception of natural object shape. Perception & Psychophysics 66: 342–351. 15129753 10.3758/bf03194883

[15] Cooke T, Jäkel F, Wallraven C, Bülthoff HH (2007) Multimodal similarity and categorization of novel, three-dimensional objects. Neuropsychologia 45: 484–495 doi:10.1016/j.neuropsychologia.2006.02.009. 16580027 10.1016/j.neuropsychologia.2006.02.009

[16] Gaissert N, Wallraven C (2012) Categorizing natural objects: a comparison of the visual and the haptic modalities. Experimental Brain Research 4: 123–134 doi:10.1007/s00221–011–2916–4. 10.1007/s00221-011-2916-4 22048319

[17] Gaissert N, Wallraven C, Bülthoff HH (2010) Visual and haptic perceptual spaces show high similarity in humans. Journal of Vision 10: 1–20 doi:10.1167/10.11.2. 10.1167/10.11.2 20884497

[18] Ernst MO, Banks MS (2002) Humans integrate visual and haptic information in a statistically optimal fashion. Nature 415: 429–433 doi:10.1038/415429a. 11807554 10.1038/415429a

[19] Op De Beeck HP, Torfs K, Wagemans J (2008) Perceived shape similarity among unfamiliar objects and the organization of the human object vision pathway. Journal of Neuroscience 28: 10111–10123. 18829969 10.1523/JNEUROSCI.2511-08.2008 PMC6671279

[20] Lacey S, Tal N, Amedi A, Sathian K (2009) A putative model of multisensory object representation. Brain Topography 21: 269–274. 19330441 10.1007/s10548-009-0087-4 PMC3156680

[21] Newell FN, Ernst MO, Tjan BS, Bülthoff HH (2001) Viewpoint dependence in visual and haptic object recognition. Psychological Science 12: 37–42. 11294226 10.1111/1467-9280.00307

[22] Craddock M, Lawson R (2010) The effects of temporal delay and orientation on haptic object recognition. Attention, Perception, & Psychophysics 72: 1975–1980. 10.3758/APP.72.7.1975 20952793

[23] Lawson R (2011) An investigation into the cause of orientation-sensitivity in haptic object recognition. Seeing and Perceiving 24: 293–314. 21864466 10.1163/187847511X579052

[24] James TW, Kim S, Fisher JS (2007) The neural basis of haptic object processing. Canadian Journal of Experimental Psychology/Revue canadienne de psychologie expérimentale 61: 219–229 doi:10.1037/cjep2007023. 17974316 10.1037/cjep2007023

[25] Miquée A, Xerri C, Rainville C, Anton JL, Nazarian B, et al (2008) Neuronal substrates of haptic shape encoding and matching: a functional magnetic resonance imaging study. Neuroscience 152: 29–39 doi:10.1016/j.neuroscience.2007.12.021. 18255234 10.1016/j.neuroscience.2007.12.021

[26] Lederman SJ, Klatzky RL (1990) Haptic object classification of common objects: Knowledge driven exploration. Cognitive Psychology 22: 421–459. 2253454 10.1016/0010-0285(90)90009-s

[27] Haag S (2011) Effects of vision and haptics on categorizing common objects. Cognitive Processing 12: 33–39. 20721600 10.1007/s10339-010-0369-5

[28] Regier T, Kay P (2009) Language, thought, and color: Whorf was half right. Trends in Cognitive Sciences 13: 439–446 doi:10.1016/j.tics.2009.07.001. 19716754 10.1016/j.tics.2009.07.001

[29] Schwarzer G, Küfer I, Wilkening F (1999) Learning categories by touch: on the development of holistic and analytic processing. Memory & Cognition 27: 868–877. 10540815 10.3758/bf03198539

[30] Homa D, Kahol K, Tripathi P, Bratton L, Panchanathan S (2009) Haptic concepts in the blind. Attention, Perception, & Psychophysics 71: 690–698. 10.3758/APP.71.4.690 19429952

[31] Gaissert N, Bülthoff HH, Wallraven C (2011) Similarity and categorization: From vision to touch. Acta Psychologica 138: 219–230 Available: http://www.sciencedirect.com/science/article/pii/S0001691811001302. 21752344 10.1016/j.actpsy.2011.06.007

[32] Do P, Homa D, Ferguson R, Crawford T (2012) Haptic Concepts. In: El Saddik A, editor. Haptics Rendering and Applications. InTech. 3–24.

[33] Lederman SJ, Klatzky RL (2009) Haptic perception: a tutorial. Attention, Perception, & Psychophysics 71: 1439–1459 doi:10.3758/APP. 10.3758/APP.71.7.1439 19801605

[34] Riesenhuber M, Poggio T (2002) Neural mechanisms of object recognition. Current Opinion in Neurobiology 12: 162–168. 12015232 10.1016/s0959-4388(02)00304-5

[35] Giese MA, Poggio T (2003) Neural mechanisms for the recognition of biological movements. Nature Reviews Neuroscience 4: 179–192 doi:10.1038/nrn1057. 12612631 10.1038/nrn1057

[36] Damper RI, Harnad SR (2000) Neural network models of categorical perception. Perception & Psychophysics 62: 843–867. 10883589 10.3758/bf03206927

[37] Liebenthal E, Binder JR, Spitzer SM, Possing ET, Medler D (2005) Neural substrates of phonemic perception. Cerebral Cortex 15: 1621–1631 doi:10.1093/cercor/bhi040. 15703256 10.1093/cercor/bhi040

[38] Regan DM (1987) Evoked Potentials and Colour-Defined Categories. In: Harnad S, editor. Categorical Perception: The Groundwork of Cognition. New York, US: Cambridge University Press. 444–454.

